# Supplementary material for: Invadopodia enable cooperative invasion and metastasis of breast cancer cells
Source: Commun Biol. 2022 Aug 1;5:758. doi: 10.1038/s42003-022-03642-z (PMC9343607; doi:10.1038/s42003-022-03642-z)
Supplement: Supplementary file 3 — Description of Additional Supplementary Files [file 42003_2022_3642_MOESM3_ESM.pdf]

Description of Additional Supplementary Materials

Invadopodia enable cooperative invasion and metastasis of breast cancer cells

L. Perrin, E. Belova, B. Bayarmagnai, E. Tüzel, B. Gligorijevic*

*Corresponding author. Email: bojana.gligorijevic@temple.edu

Additional Supplementary Materials for this manuscript include the following:

Movies S1 to S10 (.avi)

Supplementary Data 1 (.xls)

File name: Movie S1.
Description: Time lapse of a 4T1 (top panel) and a 67NR (bottom panel) monolayer in the scratch assay. Time is in hh:mm. Scale bar: 100 µm.

File name: Movie S2.
Description: Confocal z stack of a mixed 4T1-mScarlet (magenta) and 67NR-GFP (green) spheroid grown in a 3D collagen I matrix for 3 days. Scale bar: 100 µm.

File name: Movie S3.
Description: Time lapse of 4T1-mScarlet cells in a mixed spheroid embedded in collagen I. Spheroids were treated from day 0 with DMSO control. 67NR-GFP cells are not shown to ease visualization. The left panel shows all trajectories, the middle panel shows a representative core trajectory (from edge to core) and the right panel shows a representative edge trajectory (from edge to edge). Time is in hh:mm. Scale bar: 100 µm.

File name: Movie S4.
Description: Time lapse of 67NR-GFP cells in a mixed spheroid embedded in collagen I. Spheroids were treated from day 0 with DMSO control. 4T1-mScarlet cells are not shown to ease visualization. The left panel shows all trajectories, the middle panel shows a representative core trajectory (from core to edge) and the right panel shows a representative edge trajectory (from edge to edge). Time is in hh:mm. Scale bar: 100 µm.

File name: Movie S5.
Description: Time lapse of 4T1-mScarlet cells in a mixed spheroid embedded in collagen I. Spheroids were treated from day 0 with GM6001. 67NR-GFP cells are not shown to ease visualization. The left panel shows edge trajectories and the right panel shows edge trajectories. Time is in hh:mm. Scale bar: 100 µm.

File name: Movie S6.
Description: Time lapse of 67NR-GFP cells in a mixed spheroid embedded in collagen I. Spheroids were treated from day 0 with GM6001. 4T1-mScarlet cells are not shown to ease visualization. The left panel shows edge trajectories and the right panel shows edge trajectories. Time is in hh:mm. Scale bar: 100 µm.

File name: Movie S7.
Description: Time lapse of 4T1-mScarlet cells in a mixed spheroid embedded in agarose. 67NR-GFP cells are not shown to ease visualization. Representative trajectories are shown. Time is in hh:mm. Scale bar: 100 µm.

File name: Movie S8.
Description: Time lapse of 4T1-mScarlet cells in a mixed spheroid embedded in agarose. 67NR-GFP cells are not shown to ease visualization. Representative edge trajectories are shown to illustrate the inward movement of cells. Time is in hh:mm. Scale bar: 100 µm.

File name: Movie S9.
Description: Time lapse of 4T1-mScarlet cells in a mixed spheroid embedded in agarose. 67NR-GFP cells are not shown to ease visualization. A representative core trajectory is shown to illustrate a cell reaching then leaving the edge compartment. Time is in hh:mm. Scale bar: 100 µm.

File name: Movie S10.
Description: Time lapse of 4T1-mScarlet (gray) cells at the gelatin/poly-L-lysine interface (red line). A cell that crossed the interface and migrated back to the gelatin layer is indicated with a yellow arrowhead. Time is in hh:mm. Scale bar: 50 µm.

File name: Supplementary Data 1
Description: Source data of the experimental variables measured in this study as well as the metrics and statistics used. Each tab, from Fig. 1a to Fig. S12f corresponds to a figure panel. FOV, field of view; SEM, standard error of the mean.
