# Supplementary material for: Invadopodia enable cooperative invasion and metastasis of breast cancer cells
Source: Commun Biol. 2022 Aug 1;5:758. doi: 10.1038/s42003-022-03642-z (PMC9343607; doi:10.1038/s42003-022-03642-z)
Supplement: Supplementary file 15 — Reporting Summary [file 42003_2022_3642_MOESM15_ESM.pdf]

nature portfolio

Corresponding author(s): Bojana Gligorijevic

Last updated by author(s): 05/10/2022

Reporting Summary

Nature Portfolio wishes to improve the reproducibility of the work that we publish. This form provides structure for consistency and transparency in reporting. For further information on Nature Portfolio policies, see our Editorial Policies and the Editorial Policy Checklist.

Statistics

For all statistical analyses, confirm that the following items are present in the figure legend, table legend, main text, or Methods section.

n/a	Confirmed	
☐	☒	The exact sample size (*n*) for each experimental group/condition, given as a discrete number and unit of measurement
☐	☒	A statement on whether measurements were taken from distinct samples or whether the same sample was measured repeatedly
☐	☒	The statistical test(s) used AND whether they are one- or two-sided<br>*Only common tests should be described solely by name; describe more complex techniques in the Methods section.*
☒	☐	A description of all covariates tested
☐	☒	A description of any assumptions or corrections, such as tests of normality and adjustment for multiple comparisons
☐	☒	A full description of the statistical parameters including central tendency (e.g. means) or other basic estimates (e.g. regression coefficient) AND variation (e.g. standard deviation) or associated estimates of uncertainty (e.g. confidence intervals)
☒	☐	For null hypothesis testing, the test statistic (e.g. *F*, *t*, *r*) with confidence intervals, effect sizes, degrees of freedom and *P* value noted<br>*Give P values as exact values whenever suitable.*
☒	☐	For Bayesian analysis, information on the choice of priors and Markov chain Monte Carlo settings
☒	☐	For hierarchical and complex designs, identification of the appropriate level for tests and full reporting of outcomes
☒	☐	Estimates of effect sizes (e.g. Cohen's *d*, Pearson's *r*), indicating how they were calculated

Our web collection on statistics for biologists contains articles on many of the points above.

Software and code

Policy information about availability of computer code

Data collection	Data collection was done using Fluoview, C-DiGit, ImageJ 1.53c, with help of custom macros and TrackMate 6.0.1 plugin
Data analysis	Data analysis was done using Origin Pro 2021 b, Microsoft Excel 2103, R Studio 1.4.1103, Matlab 2021a with custom codes, ImageJ 1.53c with help of custom macros and TrackMate 6.0.1 plugin

For manuscripts utilizing custom algorithms or software that are central to the research but not yet described in published literature, software must be made available to editors and reviewers. We strongly encourage code deposition in a community repository (e.g. GitHub). See the Nature Portfolio guidelines for submitting code & software for further information.

Data

Policy information about availability of data

All manuscripts must include a data availability statement. This statement should provide the following information, where applicable:

- Accession codes, unique identifiers, or web links for publicly available datasets
- A description of any restrictions on data availability
- For clinical datasets or third party data, please ensure that the statement adheres to our policy

The datasets generated during and/or analysed during the current study are available from the corresponding author on reasonable request

Field-specific reporting

Please select the one below that is the best fit for your research. If you are not sure, read the appropriate sections before making your selection.

☒ Life sciences ☐ Behavioural & social sciences ☐ Ecological, evolutionary & environmental sciences

For a reference copy of the document with all sections, see nature.com/documents/nr-reporting-summary-flat.pdf

Life sciences study design

All studies must disclose on these points even when the disclosure is negative.

Sample size	Sample sizes were chosen to provide satisfactory p-values between the control and analyzed phenotypes
Data exclusions	No data was excluded from analyses
Replication	All attempts at replication were successful
Randomization	Randomization is not relevant to the study
Blinding	Blinding is not relevant to this study

Reporting for specific materials, systems and methods

We require information from authors about some types of materials, experimental systems and methods used in many studies. Here, indicate whether each material, system or method listed is relevant to your study. If you are not sure if a list item applies to your research, read the appropriate section before selecting a response.

Materials & experimental systems

n/a	Involved in the study	
☐	☒	Antibodies
☐	☒	Eukaryotic cell lines
☒	☐	Palaeontology and archaeology
☐	☒	Animals and other organisms
☒	☐	Human research participants
☒	☐	Clinical data
☒	☐	Dual use research of concern

Methods

n/a	Involved in the study	
☒	☐	ChIP-seq
☒	☐	Flow cytometry
☒	☐	MRI-based neuroimaging

Antibodies

Antibodies used	Anti-collagen I ¾ (immunoGlobe, 0207-050), anti E-cadherin (Invitrogen, 13-1900), anti N-cadherin (BD Transduction Laboratories, 610920) and anti-Tks5 (Millipore, Clone 13H6.3, Cat. # MABT336, Lot # 3068045)
Validation	-The anti-collagen I ¾ was validated by localization, with the negative control where MMP pan-inhibitor GM6001 was used in multiple papers and by several laboratories. We have previously used the same lot in several publications (Gligorijevic et al, J. Cell Sci., vol. 125, 2012; Tonisen et al Eur. J. Cell Sci 2017; Bayarmagnai et al J. Cell Sci 2019).<br>-E-cadherin and N-cadherin were validated via 4T1/67NR pair expression in Western Blots, and their localization in immunofluorescence; 4T1 is E-cad +, N-cad - and vice versa for 67NR cells (Lou et al, Dev. Dyn., 237: 2755-2768, 2008). Our data are in line with these results (Supp fig 2A). Certificate of analysis is available upon request.<br>-The anti-Tks5 antibody was validated by comparing WT cells to Tks5 KD in western blot (Figure 5A). Certificate of analysis is available here: https://www.emdmillipore.com/US/en/product/Anti-TKS5-Antibody-clone-13H6.3,MM_NF-MABT336?bd=1#anchor_COA

Eukaryotic cell lines

Policy information about cell lines

Cell line source(s)	4T1 and 67NR were purchased from Karmanos Institute, and MDA-MB-231 from ATCC
Authentication	None of the cell lines were authenticated
Mycoplasma contamination	All cell lines tested negative for mycoplasma contamination
Commonly misidentified lines (See ICLAC register)	None of the cell lines were commonly misidentified lines

Animals and other organisms

Policy information about studies involving animals; ARRIVE guidelines recommended for reporting animal research

Laboratory animals	Female mice, 5-7 weeks of age, Balb/cJ
Wild animals	N/A
Field-collected samples	N/A
Ethics oversight	All protocols were approved by Temple University IACUC

Note that full information on the approval of the study protocol must also be provided in the manuscript.
